# Bilinguals’ speech perception in noise: Perceptual and neural associations

**DOI:** 10.1371/journal.pone.0264282

**Published:** 2022-02-23

**Authors:** Dana Bsharat-Maalouf, Hanin Karawani

**Affiliations:** Department of Communication Sciences and Disorders, University of Haifa, Haifa, Israel; All India Institute of Speech and Hearing, INDIA

## Abstract

The current study characterized subcortical speech sound processing among monolinguals and bilinguals in quiet and challenging listening conditions and examined the relation between subcortical neural processing and perceptual performance. A total of 59 normal-hearing adults, ages 19–35 years, participated in the study: 29 native Hebrew-speaking monolinguals and 30 Arabic-Hebrew-speaking bilinguals. Auditory brainstem responses to speech sounds were collected in a quiet condition and with background noise. The perception of words and sentences in quiet and background noise conditions was also examined to assess perceptual performance and to evaluate the perceptual-physiological relationship. Perceptual performance was tested among bilinguals in both languages (first language (L1-Arabic) and second language (L2-Hebrew)). The outcomes were similar between monolingual and bilingual groups in quiet. Noise, as expected, resulted in deterioration in perceptual and neural responses, which was reflected in lower accuracy in perceptual tasks compared to quiet, and in more prolonged latencies and diminished neural responses. However, a mixed picture was observed among bilinguals in perceptual and physiological outcomes in noise. In the perceptual measures, bilinguals were significantly less accurate than their monolingual counterparts. However, in neural responses, bilinguals demonstrated earlier peak latencies compared to monolinguals. Our results also showed that perceptual performance in noise was related to subcortical resilience to the disruption caused by background noise. Specifically, in noise, increased brainstem resistance (i.e., fewer changes in the fundamental frequency (F0) representations or fewer shifts in the neural timing) was related to better speech perception among bilinguals. Better perception in L1 in noise was correlated with fewer changes in F0 representations, and more accurate perception in L2 was related to minor shifts in auditory neural timing. This study delves into the importance of using neural brainstem responses to speech sounds to differentiate individuals with different language histories and to explain inter-subject variability in bilinguals’ perceptual abilities in daily life situations.

## Introduction

Daily interactions frequently occur in acoustically challenging environments, which often pose challenges to listeners. One challenging listening condition that deteriorates speech perception is background noise [1–3]. It is well-known that speech perception in the presence of background noise can be more challenging for bilinguals compared to monolinguals [4–16] and that this disadvantage is present even when bilinguals have early exposure and strong proficiency in both languages [7, 16, 17]. At the same time, the literature notes the advantages of bilingualism for brain structure and function (e.g., [18, 19]). Specifically, electrophysiological tests that examine subcortical processes show enhanced auditory processes in bilinguals (e.g., [20–28]). To date, the puzzling question of how the perceptual disadvantage of bilinguals aligns with the advantage seen in subcortical processes remains. In an attempt to answer this question, the current study examined the association between perceptual and physiological outcomes.

The auditory brainstem response evoked by speech stimuli is used to examine how subcortical structures of the auditory pathway encode temporal and spectral aspects of speech sounds (e.g., [29–31]). The frequency-following response (FFR) evoked by these speech stimuli can closely mimic the waveform of the acoustic stimulus [32]. The FFR has been studied in bilingual populations to examine whether lifelong language experience can affect subcortical auditory processing. Enhancements in FFRs because of bilingualism were reflected in different aspects of the neural response. For example, some studies showed greater consistency and stability of FFRs among bilinguals compared to monolinguals [25, 28, 33] and among bilinguals who are more proficient in the language [25] and have more years of bilingual experience [27]. Other studies showed that exposure to a second language induces earlier neural latencies [20], more pronounced and robust FFRs [22, 23], and a larger representation of the fundamental frequency (F0) component [24, 26, 27], which is used to recognize and track speech, and serves as an important cue for speech perception in challenging listening conditions [26, 34–38]. Notably, differences between monolinguals and bilinguals in FFRs were observed mainly when the speech stimulus was presented in background noise compared to quiet conditions [e.g., 20, 24]. Further, better representation of F0 among bilinguals was associated with better attentional and cognitive abilities, mainly when individuals were tested in noise (e.g., [24, 25]).

To the best of our knowledge, this study is the first to combine perceptual and brain measures in bilingual populations. This combination is important for translating knowledge regarding physiological outcomes into practice and for examining how the advantage in subcortical responses interacts with a disadvantage in perception. Further, in the current study, we examined how the perceptual-physiological correlation varies across the two languages of bilinguals. For this purpose, in addition to comparing the perceptual performance of bilinguals and monolinguals, bilinguals were examined in both languages: (first language—L1 (Arabic) and second language—L2 (Hebrew)). This was done to determine whether the subcortical physiological mechanism can predict bilinguals’ perceptual performance in general or related to the language of the processed stimuli.

In summary, the current study was designed to answer the primary research questions: Does subcortical processing predict perceptual performance? And how do L1 and L2 modify the perceptual-physiological correlation?

## Materials and methods

### Participants

Sixty, right-handed, first-year college students (41 females, mean age = 24.6 ± 3.7) were recruited to participate in the study. All had obtained a similar level of formal education (mean ± standard deviation = 13.3 ± 2.6 years) and had normal cognitive function (based on Wechsler Intelligence Test [39]). In addition, all participants exhibited normal hearing thresholds in both ears (≤ 20 dB HL pure-tone air conduction thresholds for octave frequencies 250 through 8000 Hz [40]) and absolute peak and interpeak latencies within normal limits [41] to 100-μs clicks presented at 80 dB nHL at a rate of 13.3/s, with a 10.66 ms recording window. To control for knowledge of music, a factor known to affect subcortical processing (e.g., [42–50]), only participants with no- to minimal- musical knowledge (less than one year of experience in elementary school) were included. Professional musicians were excluded from the study. Participants provided written informed consent before participating and were compensated with either a coffee coupon or course credit for participating. The Ethics Committee of the University of Haifa approved the study protocol.

Participants were divided into two groups based on their language history and knowledge: Arabic-Hebrew bilinguals and Hebrew monolinguals. All participants were asked to fill out a questionnaire about their demographic information and language profile [adapted from 12, 51]. The information reported below is based on self-reports. The means and percentages reported are based on group averages.

The bilingual group consisted of 30 Arabic-Hebrew speakers. Bilinguals were exposed to Arabic (L1) from birth and to Hebrew (L2) from age three. All bilinguals considered themselves dominant in L1 and reported intensive exposure to this language. Bilinguals received more than 10 years of formal academic education in Hebrew (12.3 ± 3.9 years), passed the high school matriculation exams with Hebrew as their L2, and used Hebrew extensively in their academic studies (about 45% of the time). The monolingual group consisted of 30 participants who reported only knowledge of Hebrew and had no substantial learning or proficiency in Arabic.

Data of one monolingual participant were excluded because of excessive noise in the electrophysiological recordings caused by high levels of myogenic activity. Consequently, data from 59 participants (30 bilinguals and 29 monolinguals) were included in the final analysis. Except for language history and knowledge, the two groups were similar in chronological age (*t* (57) = -1.857, *p =* 0.07), gender (*t* (57) = -0.085, *p =* 0.933) and years of formal education (*t* (57) = 1.529, *p =* 0.132). All participants underwent an electrophysiological recording and performed perceptual tasks in the hearing lab at the University of Haifa.

### Electrophysiology

During the electrophysiological session, participants sat in a comfortable reclining chair in a sound-treated, electrically shielded booth and were asked to stay calm during passive exposure to the stimuli. Lights inside the audiological booth were dimmed during recording. Brainstem responses were collected using the Biologic Navigator Pro System (Natus Medical Inc., Mundelein, USA). Vertical montage for Ag-AgCl electrode placement was applied. The non-inverting electrode was at the midline (Cz), the negative inverting electrode on the right earlobe (A2), and the ground electrode was put on the left earlobe (A1). The maximum permissible impedance level for each electrode was less than 5 kΩ, and the inter-electrode impedance was less than 3 kΩ.

#### Stimuli and conditions

A 40-ms synthesized /da/ syllable was used. This universal syllable was chosen since it is shared across many languages [52], including Arabic and Hebrew, and was previously used in Karawani and Banai [53] with Arabic and Hebrew speakers. This five-formant synthesized speech syllable is comprised of an initial noise burst, followed by a formant transition between the consonant [d] and the vowel [a]. The syllable contains the fundamental frequency [F0] that linearly rises from 103 to 125 Hz and five additional formant frequencies [refer to 53, 54 for a detailed description regarding the stimulus characteristics and formants]. This syllable was presented at 80 dB SPL at a rate of 10.9 Hz, with an alternating polarity to minimize cochlear microphonics and stimulus artifacts [55]. The recording window was 85.33 ms, including a pre-stimulus period of 15 ms. Brainstem responses were elicited in response to the speech syllable in quiet and in noise conditions. In noise, the syllable was presented with 80 dB SPL continuous, white noise with a signal-to-noise ratio (SNR) of 0 dB. The stimuli in the two listening conditions were presented monaurally to the right ear via electromagnetically shielded biologic insert earphones (580-SINSER) while leaving the left ear unoccluded. Similar to the protocol used in Krizman et al. [54], all participants in the current study heard a movie soundtrack played at < 40 dB SPL (an insufficient intensity to mask the stimulus in the right ear) with the unoccluded ear. This was done to promote stillness and rule out differences in state as a potential confound.

#### Recording

Two blocks of 3000 artifact-free sweeps were collected in each of the two listening conditions. Trials with activity exceeding ± 25 μV were rejected. Responses were online band-pass filtered from 100 to 2000 Hz, which captures the limits of the brainstem and the inferior colliculus phase-locking and minimizes collecting myogenic noise and cortical activities [29, 55, 56]. The total recording time was 20 minutes.

#### Data averaging and analysis

A final waveform was created for each listening condition by averaging the two blocks collected. The final waveform comprised 6000 artifact-free sweeps. In total, two final averaged waveforms were analyzed for each participant, one for the quiet condition and the other for the noise condition. Transient peaks and those reflecting the harmonic portion of the stimulus were visually identified and manually marked. Detailed information regarding the FFR components is described in previous reports (e.g., [31, 32, 57]). In this study, all components (V, A, C, D, E, F, and O peaks) were detected in the quiet condition. However, we considered analysis of the V, A, and O peaks reflecting the initiation and the offset of the response, and peak F corresponding to the voicing of the speech sound. We focused solely on these peaks because the detectability of the remaining peaks (C, D, and E) was relatively poor in the noise condition (64.4%, 66.1%, and 76.2%, respectively). A peak was considered reliable if it was present in >85% of participants [58]. Peaks of interest (V, A, F, and O) were identified by the two authors. The second author marked the waveforms separately to verify uniform marking and was also blinded to participants’ identities and group. In addition, to avoid bias, the second author was blinded to the condition under which the recording was conducted. Measures of both timing (latency) and magnitude (amplitude) were applied for peaks V, A, F, and O. Also, a fast Fourier transform (FFT) in Matlab (The Mathworks) using the Brainstem Toolbox [31] was performed to calculate F0 amplitude.

### Perception

In this part, bilinguals were tested with Arabic (L1) and Hebrew (L2) speech stimuli, with language order counterbalanced, while monolinguals were examined only in Hebrew. Bilinguals were given a 10-minute break between Arabic and Hebrew perceptual tasks. Here, we examined the ability of participants to perceive wordlists and sentences presented in quiet and with background noise (detailed below). Participants performed the perception part individually while sitting in front of a laptop. Participants were asked to listen to a given stimulus and repeat it. This procedure continued until all stimuli had been presented. Each participant heard each stimulus only once, no stimulus was used twice, and no feedback was provided. The presentation order of the speech stimuli (wordlists and sentences) and listening conditions (quiet and noise) was randomized across participants.

#### Stimuli and conditions

Participants were presented with two types of speech stimuli (wordlists and sentences), which differed in the contextual cues included. At the wordlists level, words from audiological speech and hearing tests were used to compose each wordlist [59]. Seven bi-syllabic words from unrelated semantic categories were used in each wordlist. At the level of the sentences, plausible, syntactically correct sentences from Arabic and Hebrew versions of the Hagerman test [60] were adapted and used. Each sentence consisted of seven words with semantic and syntactic redundancies.

Instead of relying on one type of speech stimulus, we included two types to reflect the perception of the individual. This was done because previous studies have shown that bilinguals show more difficulty in noise as the linguistic complexity of the task increases [7, 17, 61–67]. Here, to evaluate the relation between physiology and perceptual measures, we combined the perceptual accuracy of the individual in both wordlists and sentences to reflect the perceptual performance in general, regardless of the effect of task complexity on performance.

Wordlists and sentences in Arabic and Hebrew were created. The average number of syllables across Arabic and Hebrew stimuli was similar (*p* > 0.19), as were the root-mean-square (RMS) amplitudes (*p* > 0.7). Stimuli across the two languages were also similar in terms of frequency (tested in a pilot study). Uncommon words, cognates and false cognates were not included in the data set. An Arabic speaker recorded stimuli in Arabic, and a Hebrew speaker recorded Hebrew stimuli to avoid bias resulting from pronunciation problems.

Stimuli were presented binaurally to participants at 80 dB SPL in two listening conditions: quiet and a 4-talker, babble noise at a fixed level of SNR = 0 dB. For the noise condition, the babble used for Arabic stimuli was in Arabic, and the babble used for Hebrew stimuli was in Hebrew. The babble noise was selected because it closely resembles a natural situation where individuals need to extract a target speech from a background of competing voices. We assessed and normalized the amplitudes of the Arabic and Hebrew babbles to ensure equal intensity and analyzed a set of acoustic parameters to reveal similar babble characteristics across languages [68]. Stimuli were presented to participants through software developed and used previously [69].

#### Scoring

Participants’ perceptual responses were digitally audio-recorded using a Mini USB recorder. Two trained coders who were blind to the study goals coded each participant’s responses. One point was given for each word that the participant could repeat. The perceptual accuracy for each condition was examined. This was done by calculating the percentage of correct responses accomplished over all wordlists and sentences given in each listening condition.

### Statistical analysis

Data from 59 participants (30 bilinguals and 29 monolinguals) are reported below and were included in the final analysis. We focused on condition (quiet vs. noise) and group (monolinguals vs. bilinguals) differences in the electrophysiological analysis. Dependent variables included the latencies and amplitudes of V, A, F, and O peaks and F0 amplitude. In the perceptual analysis, within participant comparisons were also conducted (bilingual L1 vs. L2) as a main effect of language. All statistical analyses were completed using IBM SPSS Statistics V25. Repeated measures analyses of variance (ANOVA) were performed. Pairwise comparisons were used when required. All statistical analyses were adjusted for multiple comparisons using Bonferroni corrections [70], and effect sizes were indicated using *η*^*2*^*p*. Shapiro-Wilk tests were used to test for normal distribution within each group. Levene’s tests were conducted to test the homogeneity of variance for all measures. Finally, Pearson *r* correlations coefficient were calculated to study the perceptual-physiological association. Detailed analyses are presented in the Results subsections below.

## Results

### Electrophysiology

Repeated measures ANOVA with condition (quiet, noise) as the within participant factor and group (monolingual, bilingual) as a between participant factor was used.

#### Latency

**Fig 1:**
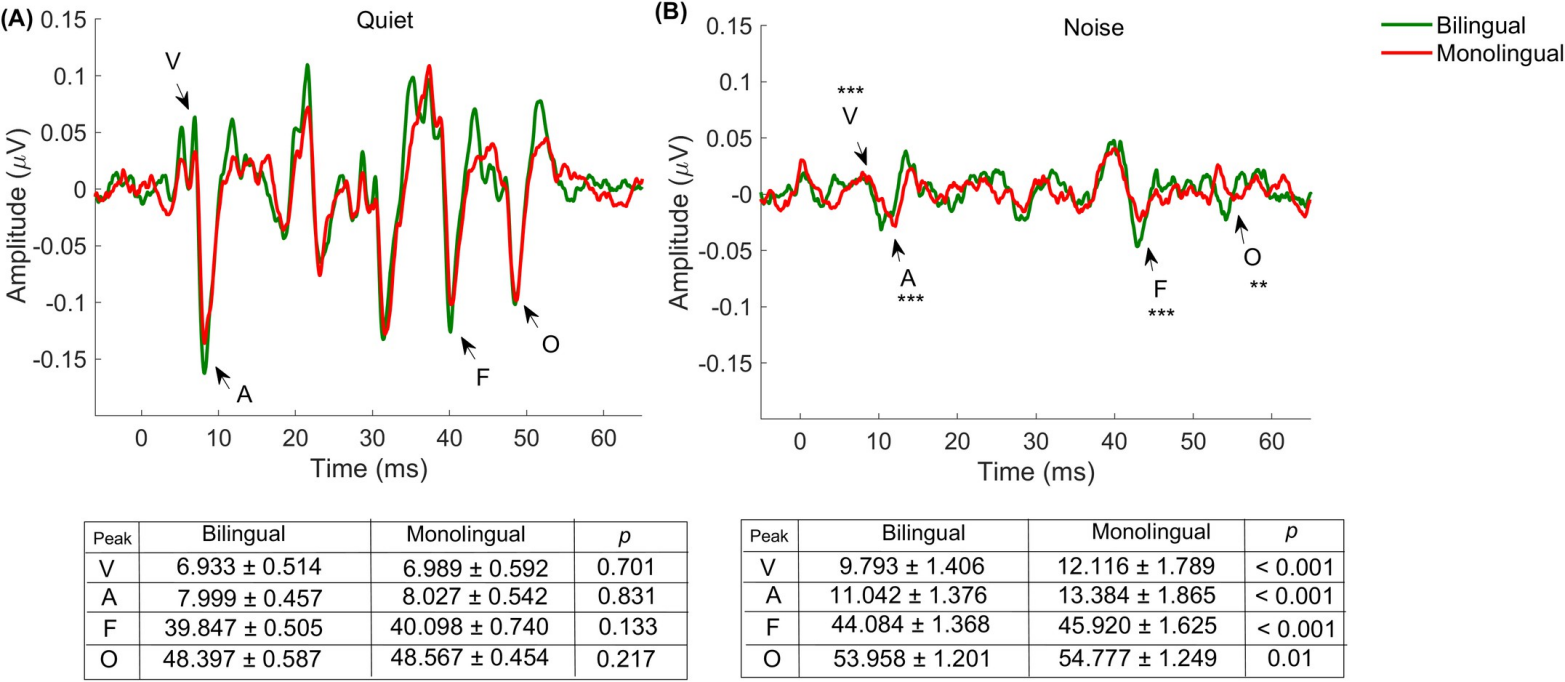
Grand average subcortical responses to speech stimuli obtained from bilinguals (green) and monolinguals (red) recorded in quiet **(A)** and noise **(B)**. ** *p* ≤ 0.01; *** *p* ≤ 0.001. Significant group differences between peak latencies (as revealed in *t* tests for independent samples) were found in noise. The tables below represent means ± SD (ms) for peak latencies in quiet (right) and noise (left), and the *p* value for the group differences.

Main effects of condition and group were observed for peaks V, A, F, and O (see Table 1). Bonferroni pairwise comparisons revealed that latencies were significantly prolonged in noise compared to quiet (*p* values < 0.001), and significantly earlier latencies were observed in the bilingual group compared to the monolingual group (*p* ≤ 0.005). Significant condition × group interactions were observed for all peak latencies (*p* ≤ 0.01). These significant interactions indicate that the presence of noise prolonged the peak latencies in one group to a greater extent than in the other. Specifically, *post hoc t* tests showed that whereas the latencies of the peaks were comparable in quiet (see Fig 1A, *p* ≥ 0.133), earlier latencies were observed in the bilingual group compared to the monolingual group in noise. The prolonged latencies of bilinguals compared to monolinguals in noise are illustrated in Fig 1B (*p* ≤ 0.01).

**Table 1:** Effects of condition and group.

		Latency			Amplitude		
	Peak/Component	*F*	*P*	*η* ^*2*^ *p*	*F*	*p*	*η* ^*2*^ *p*
**Condition**	**V**	304.944	<0.001***	0.843	28.670	<0.001***	0.335
	**A**	354.953	<0.001***	0.862	191.241	<0.001***	0.770
	**F**	537.149	<0.001***	0.904	34.266	<0.001***	0.375
	**O**	1108.214	<0.001***	0.951	70.557	<0.001***	0.553
	**F0 amplitude**	----			67.079	<0.001***	0.541
**Group**	**V**	31.041	<0.001***	0.353	2.078	0.155	0.035
	**A**	28.397	<0.001***	0.333	1.045	0.311	0.018
	**F**	25.479	<0.001***	0.309	2.839	0.1	0.047
	**O**	8.447	0.005**	0.129	2.753	0.103	0.046
	**F0 amplitude**	----			0.291	0.591	0.005
**Condition x Group**	**V**	24.566	<0.001***	0.301	1.454	0.233	0.025
	**A**	26.944	<0.001***	0.321	1.947	0.168	0.033
	**F**	13.341	0.001***	0.190	0.111	0.740	0.002
	**O**	3.366	0.01**	0.056	0.174	0.678	0.003
	**F0 amplitude**	----			0.075	0.785	0.001

Values of repeated measures ANOVA as a function of condition (quiet and noise) and group (monolingual, bilingual) for the mean latencies and amplitudes of FFR components. Degrees of freedom (between groups) = 57; (within group) = 1, partial eta square *(η*^*2*^*p*),

* *p* ≤ 0.05

** *p* ≤ 0.01;

*** *p* ≤ 0.001.

#### Amplitude

A main effect of condition was observed for peaks V, A, F, and O, and F0 amplitude (Table 1, *p* < 0.001). Background noise diminished the amplitudes of all peaks (see Fig 1) and F0 amplitude in both groups. No significant main effect of group was observed (Table 1, *p* ≥ 0.1). Condition x group interactions were also not significant (Table 1, *p* ≥ 0.168). Monolinguals and bilinguals demonstrated similar V, A, F, and O peak amplitudes in the two listening conditions, and F0 amplitudes were comparable in quiet (monolingual mean amplitude (μV) = 7.495 ± 3.422, bilingual mean amplitude (μV) = 7.936 ± 2.730) and noise (monolingual mean amplitude (μV) = 3.729 ± 2.957, bilingual mean amplitude (μV) = 3.908 ± 2.247) across the two groups.

### Perception

The perception was compared between groups (monolinguals vs. bilinguals, both operating in Hebrew) and within the bilingual group (bilinguals in L1 vs. L2).

#### Between groups

**Fig 2:**
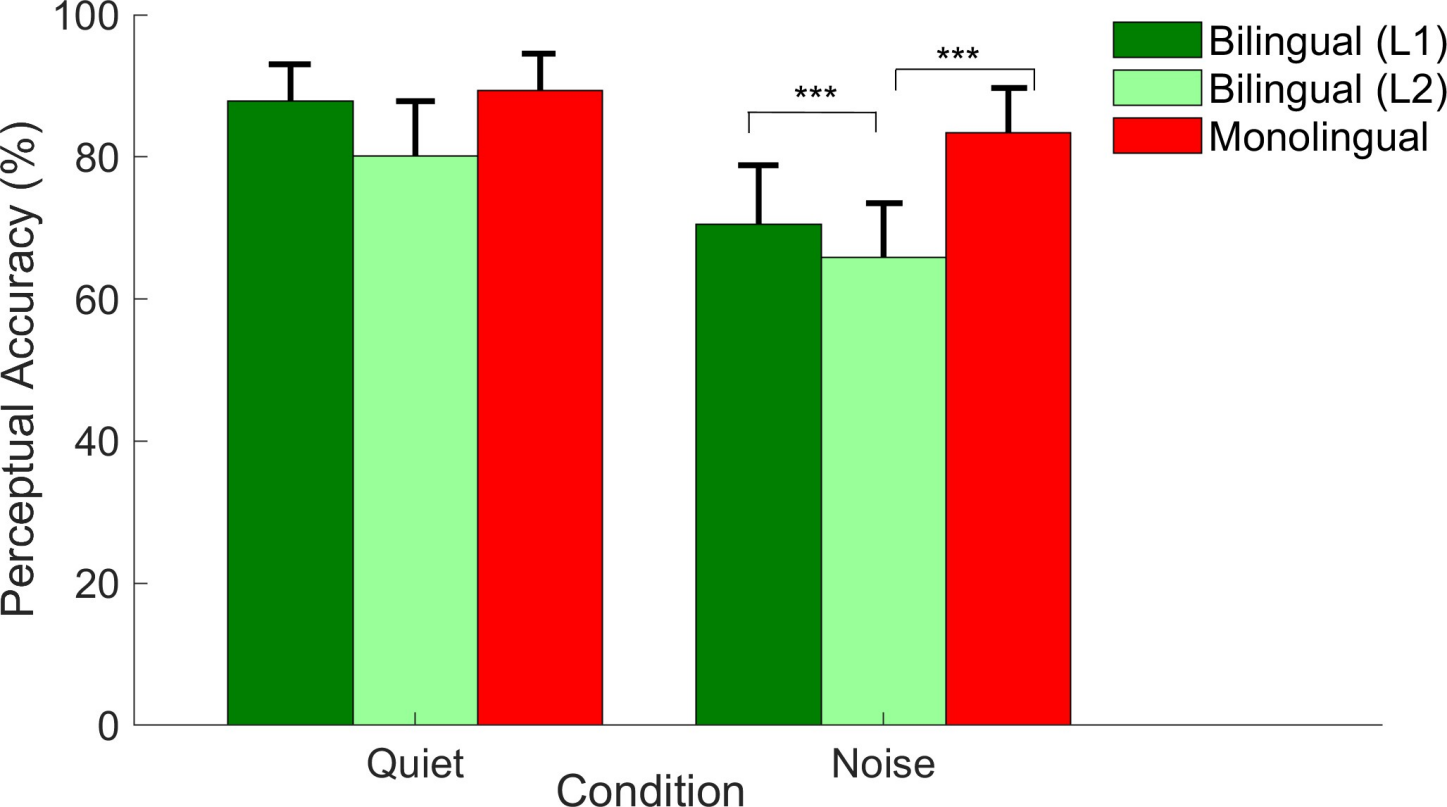
Perceptual accuracy among the bilingual group in L1 (Arabic; dark green columns), L2 (Hebrew; light green columns), and the monolingual group (Hebrew; red columns) in quiet and noise. Error bars represent the standard deviations of the mean. Asterisks denote significant differences between groups or languages. *** *p* < 0.001.

*Monolinguals vs*. *bilinguals*. Repeated measures ANOVA with condition (quiet, noise) as within participant factor and group (monolingual, bilingual (in Hebrew)) as a between participant factor was used. Main effect of condition (F (1, 57) = 643.226, *p* < 0.001, *η*^*2*^*p* = 0.919) was observed. Accuracy levels were higher in quiet compared to noise (Fig 2). Main effect of group (*F* (1, 57) = 59.525, *p* < 0.001, *η*^*2*^*p* = 0.511) and a condition x group interaction (*F* (1, 57) = 108.844, *p* < 0.001, *η*^*2*^*p* = 0.656) were significant. As shown in Fig 2, no differences were observed between groups in quiet (monolingual mean in quiet = 89.334 ± 5.217, bilingual L2 mean in quiet = 80.142 ± 7.703, *t* (57) = -5.382, *p* = 0.7), while in noise bilinguals’ perceptual performance (in L2) was lower than that of monolinguals (monolingual mean in noise = 83.386 ± 6.313, bilingual L2 mean in noise = 65.880 ± 7.656, *t* (57) = -9.595, *p* < 0.001).

#### Within the bilingual group

Repeated measures ANOVA was used with two within participant factors (condition: quiet, noise; language: L1, L2). Main effects of condition (*F* (1, 29) = 308.341, *p* < 0.001, *η*^*2*^*p* = 0.914) and language (*F* (1, 29) = 44.252, *p* < 0.001, *η*^*2*^*p* = 0.604) and a condition x language interaction (*F* (1, 29) = 5.512, *p* = 0.026, *η*^*2*^*p* = 0.160) were observed. Bilinguals had better perception in quiet than in noise. As illustrated in Fig 2, bilinguals achieved similar accuracy in L1 and L2 in quiet (mean L1 in quiet = 87.833 ± 5.210, mean L2 in quiet = 80.142 ± 7.703, *t* (29) = 7.298, *p* = 0.09) but noise had a greater effect on L2 performance, leading bilingual individuals to perform significantly poorer (*t* (29) = 3.855, *p* = 0.001) in L2 (mean = 65.880 ± 7.656) compared to L1 (mean = 70.523 ± 8.331).

### Perceptual-physiological association

To examine the perceptual-physiological association, we first calculated the delta (difference) between quiet and noise conditions in physiological and perceptual measures. The degree of change in F0 amplitude from quiet to noise (*delta F0* = F0 amplitude in quiet minus F0 amplitude in noise, [larger values indicate more deterioration]) and the shifts in V latency across the two listening conditions (*V latency shift* = V latency in noise minus V latency in quiet, [larger values indicate more shifts]) were calculated from the physiological aspect. We chose, in particular, these physiological measures based on previous literature showing robust correlations between F0 and perceptual accuracy (e.g., [34, 35, 47, 71–73]) and between neural timing and perceptual abilities [74, 75]. We chose the onset of the response specifically because of its sensitivity to the effects of adverse listening conditions [76, 77]. In perception, the deterioration in accuracy due to noise was calculated (*delta perception* = accuracy in quiet minus accuracy in noise [larger values reflect more deterioration in accuracy]).

#### Correlation between delta F0 and delta perception

**Fig 3:**
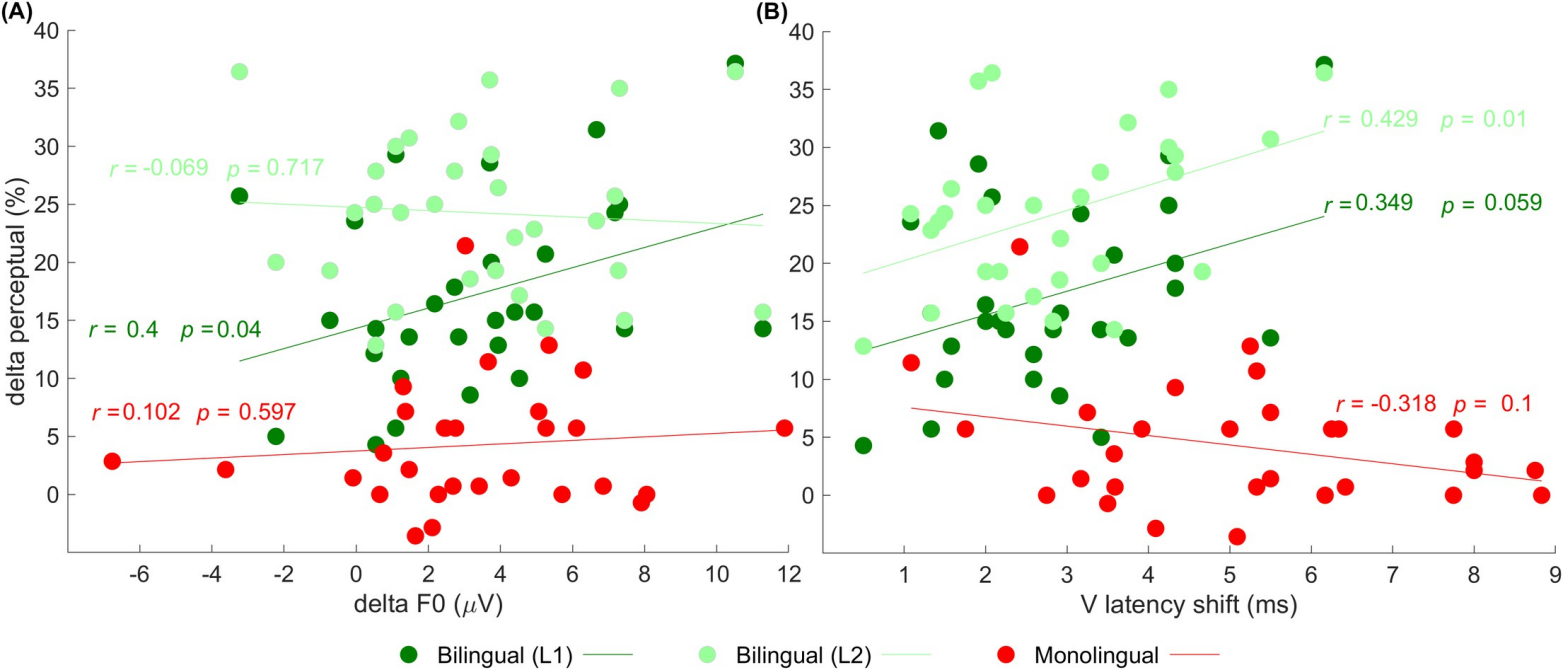
Physiological and behavioral relations. Deterioration in perceptual accuracy (delta perceptual = accuracy in quiet minus accuracy in noise; y-axis) as a function of changes in F0 amplitude (delta F0 = F0 amplitude in quiet minus F0 amplitude in noise; x-axis in (3A)) and shifts in V latency (V latency shift = V latency in noise minus V latency in quiet; x-axis in (3B)). Pearson *r* coefficient values and *p* values for bilingual L1 (dark green), bilingual L2 (light green), and for the monolingual group (red) are shown.

This correlation was conducted to test whether larger effects of noise in the neural representation (i.e., larger F0 deltas) correlated to greater susceptibility to the degradative effects of noise on the perception task (i.e., larger perception deltas). As shown in Fig 3A, this correlation was significant and positive among bilinguals in L1 (*r* = 0.4, *p* = 0.04), indicating that bilingual listeners who were more susceptible to the effect of noise and showed more significant perceptual deterioration in their dominant language, demonstrated greater susceptibility with the neural encoding of F0. This correlation was not significant among monolinguals (*r* = 0.102, *p* = 0.597) or bilinguals when tested in their L2 (*r* = -0.069, *p* = 0.717).

#### Correlation between V latency shift and delta perception

This correlation was conducted to examine whether greater changes in the latency of the physiological response due to the addition of noise are related to greater deterioration in perception. As demonstrated in Fig 3B, a significant positive correlation was found among bilinguals when tested in L2 (*r* = 0.429, *p* = 0.01), indicating that larger shifts in V latency were associated with more deterioration in accuracy. However, this correlation was not found for monolinguals (*r* = -0.318, *p* = 0.1) and was marginally significant for bilinguals when tested in their L1 (*r* = 0.349, *p* = 0.059).

## Discussion

The current study characterized the subcortical neural processing of speech sounds and the perceptual performance of normal-hearing bilinguals and monolinguals in quiet and noisy conditions. We also examined how this subcortical neural processing is related to perceptual performance. Our results demonstrated that the effect of lifelong experience with two languages–bilingualism–was reflected mainly in perceptual and neural measures in challenging listening conditions. Specifically, perceptual performance was worse for bilinguals than monolinguals in noise, but neural timing was earlier. Further, the current study showed brain-behavior associations. Among bilingual individuals, perceptual performance in noise was associated with the extent of subcortical resistance to the disruption caused by background noise. These findings are discussed in the following subsections.

### The effect of noise on perceptual accuracy and neural response

Our results indicated a considerable decline in perceptual accuracy in the presence of background noise and substantial changes in the morphology of the auditory brainstem responses. Overall, monolinguals and bilinguals (in both languages) achieved significantly lower accuracy in the noisy condition than the quiet condition, and the neural responses elicited from both groups were observed with longer latencies and smaller amplitudes in the noisy condition (Figs 1B and 2B). The poorer perceptual performance of bilinguals in L2 compared to L1 or relative to the performance of monolingual listeners is consistent with previous findings [8, 11–14, 16, 78–85] and can be attributed to multiple factors that may independently or interactively affect bilinguals’ performance, such as late age of language acquisition [7, 86, 87], lower proficiency in L2 [11, 79, 86, 88], limited exposure to the languages [16, 89–91] and co-activation between languages [92–96]. Specifically, previous studies have suggested that bilinguals tend to show perceptual difficulties in their L2 because they acquire the language at a later stage of life and exhibit low proficiency on it (e.g., [7, 11, 79, 86–88]). In addition, studies have proposed that because bilinguals split their resources across different languages, they are less exposed to each one. This limited exposure to each language may lead to less precise automatic processing and consequently pose more challenges to the listener [16, 89–91]. In addition, as bilinguals’ two languages are co-activated even when only one language is needed for the task [92–96], bilingual listeners need to manage more competing distractors during language processing. This co-activation may result in fewer resources available during speech processing and increase listening effort and perceptual difficulty [6, 16]. To note, in the current study, bilinguals showed a significant decrease in their perceptual accuracy in background noise, even when tested in their L1. The limited exposure to languages and co-activation may explain the speech perception in noise disadvantage seen in bilinguals even when operating in their dominant language [16].

From the physiological aspect, the responses elicited in noise were diminished and degraded, consistent with previous findings [30, 47, 76, 77, 97–99]. This reduction can reflect neural desynchronization [98, 100] and less efficient efferent processing [20]. Neural group differences were reflected in noise, where earlier neural latencies were observed in bilinguals compared to their monolingual peers. However, since the participants were normal-hearing, young adults with no clinical condition, it is likely that the prolonged responses observed among monolinguals do not reflect deficiencies. Instead, they indicate that the neural encoding of speech became more resistant to the detrimental effects of background noise in bilinguals. Considering differences in the linguistic experience between bilinguals and monolinguals and consistent with the evidence that FFRs are dynamic and malleable to the effects of immediate or long-term auditory experiences (e.g., [20, 24–27, 42, 44–50, 58, 101–108]), we suggest that the language experience likely underlies the group differences observed in the neural responses in the noise condition. The enriched language environment of bilinguals and their need to manage two linguistic systems may explain the early latencies observed, as these listeners become faster in detecting the characteristics of speech stimuli. Further, since bilinguals manage two linguistic systems that can compete with each other (e.g., [93, 95, 96]), these listeners are required to enhance their attention to focus on the target signals and to prevent interference from those that are irrelevant [24, 25, 109–115]. Therefore, the earlier FFR neural latencies observed in bilinguals may reflect their advantage in attentional control, mainly as FFRs are known to be sensitive to the effects of these skills [24, 25, 116–119].

In the current study, no group differences were found in the amplitudes of the neural response peaks or in the magnitude of the fundamental frequency. These findings are consistent with a recent study [20] suggesting that the effects of bilingualism are not detectable by measuring amplitude. However, in contrast to our results, other previous studies showed differences between monolinguals and bilinguals in the magnitudes of F0 and suggested that bilinguals encode F0 more robustly than monolinguals do (e.g., [24–26]). Several factors may explain the inconsistent findings. Some can be related to methodological differences. Specifically, a longer syllable was used in previous studies than the one used in the current study (170 ms vs. 40 ms). Consequently, the characteristics of the stimulus differ. For example, in previous studies, the 170 ms syllable consisted of a 50 ms formant transition and a 120 ms steady-state portion. Consequently, the sustained vowel period in the 170 ms stimulus is likely to capture subtle enhancements in the FFR. However, the shorter stimulus used in the current study may have theoretically restricted us from obtaining group differences in the amplitudes of F0. Furthermore, it is possible that since noise diminished the entire response dramatically (Fig 1A versus 1B), subtle differences in amplitude measures, which are known to be more variable and less stable compared to the latency measure [120], were not observed in the current analysis. Taken together, our results do not rule out the possibility that bilinguals and monolinguals differ in the amplitude aspect. Rather, they suggest that in this specific design, differences might be confined to specific factors that should be examined in more detail.

### Perceptual-physiological associations

Regarding perceptual-physiological associations, our results demonstrated that subcortical processing played a role in the perceptual abilities of bilinguals. Our findings indicate that perception is related to the degree of subcortical resilience to the disruption caused by background noise. Specifically, in noise, increased brainstem resistance (i.e., fewer changes in F0 representation or fewer shifts in V latency) were related to better speech perception abilities among bilinguals, as indicated by less deterioration in perception (Fig 3). Alternatively stated, bilinguals who exhibited more disruption of brainstem processing in noise had more perceptual difficulties. These correlations further the understanding of the neural processes underlying perception of speech among bilinguals (especially given that these correlations were not significant in the monolingual group) and suggest that subcortical processes could be one source that explains inter-subject variability in daily challenging listening conditions.

The significant correlations observed among the bilingual listeners, who were more affected perceptually by the detrimental effect of noise than their monolingual peers, suggest that the brainstem processing (low-level information) may be exploited in conditions that are more challenging for the individual. This suggestion corresponds with the Reverse Hierarchal Theory (RHT), which states that processing starts at high-level areas; however, when the task demands increase, more reliance on lower levels is needed to search for more optimal representation [121]. Also, the correlations found mainly among bilinguals suggest greater recruitment of subcortical perceptual areas by these individuals, which aligns with the anterior-to-posterior and subcortical shift (BAPSS) model [18, 122]. The BAPSS model posits that with experience, bilinguals do not rely only on the typical regions during processing. Instead, they may recruit other areas, such as automatic subcortical or posterior regions to manage the co-activation and competition between languages.

The current correlations may be evidence of the interplay between central and peripheral processes [123]. The literature has shown that FFRs are determined by peripheral processes and affected by central processes [45, 124–127]. In the ascending track, better brainstem processing–reflected in the current study as less susceptibility to the effects of noise–may provide a good platform for higher cortical processing, which can improve perceptual performance in noise [128–130]. In the opposite view of the descending path, the associations found in the current study can also be explained by the corticofugal (top-down) system [43, 44, 46, 108, 121, 131–136] by which cortical processes that are critical for understanding distorted signals [137–143] project backward to tune structures in the auditory periphery [131, 136, 144, 145], which might enhance or modify features of the target speech subcortically. Consequently, associations between perceptual performance and subcortical neural responses can also indirectly reflect the effect of the auditory cortex. In this regard, previous studies have shown that bilingual experience increases grey matter density in various cortex regions, including executive control regions [146–149], which are essential in challenging listening conditions [150, 151]. Thus, it can be argued that bilinguals who tend to use more cortical resources in background noise may have more efficient backward processes, and consequently, their brainstem responses were found to be less susceptible to the effect of noise. Future studies that examine brainstem and cortical event-related potentials (simultaneously) and their relationship with perceptual abilities could investigate the above assumptions and shed light on the interplay between central and peripheral processes.

Within the bilingual group, the correlations found yielded interesting results. The latencies of the subcortical neural response were linked more to bilinguals’ perceptual performance in L2 and marginally in L1. At the same time, the encoding of the fundamental frequency was correlated to their perceptual performance in L1. To the best of our knowledge, these correlations are innovative and have not been reported to date. Still, they can be partially explained by the results of Tremblay, Namjoshi [152], who demonstrated that language experience affects listeners’ use of F0, a cue that is important for word segmentation and comprehension of signals masked by other interferences (e.g., [36, 37, 153]); in this case, the perception of speech embedded in noise. Also, it can be argued that since bilinguals demonstrate differences in high-level (i.e., cortical) processes in their two languages [7, 17, 61, 65, 66, 154–156], the backward pathway that may modify features subcortically (consistent with the explanation of the corticofugal effect discussed above) might also differ, leading to different perceptual-neural correlations in bilinguals’ two languages. However, future studies should examine perceptual-neural associations in other groups of bilinguals to shed light on the mechanism underlying the current findings and better understand the variability in bilinguals’ L1 and L2 associations.

No correlations were found between subcortical processing and perceptual abilities in noise among monolingual listeners in the current study. The changes in F0 amplitudes and the shifts in the latency of V peak did not predict accuracy among monolingual listeners. These results align with those of Yellamsetty and Bidelman [98], who showed that F0 amplitudes failed to predict the accuracy of listeners’ identification, but contradict with others (e.g., [34, 76, 128]) who found a relationship between subcortical neural processing and perceptual performance in noise among monolingual speakers. We suggest that the main reason for not finding a significant perceptual-neural correlation among monolinguals in the current study is that noise deteriorated their perception to a lesser extent compared to bilinguals. Consequently, it is likely that the lexical access and perception occurred rapidly and automatically [157, 158], and no reliance on lower levels was needed [113]. We hypothesize that a correlation would likely be found when testing monolinguals with a more challenging noise condition that could pose a more significant perceptual challenge, and consequently, more need for reliance on lower levels. Accordingly, Song and colleagues [35] found a significant perceptual-neural correlation in their monolingual sample. Their participants were much adversely affected by the addition of noise (the highest accuracy obtained was 75%, and the averaged accuracy was 40.56%, both lower than these of the current study). Nevertheless, this assumption needs to be tested in future studies because additional methodological factors may explain the inconsistency between the different studies. Among others, studies that found a significant correlation [34, 76, 128] used a 170 ms syllable, which differs from the one used in the current study.

### Future directions, implications, and limitations

Our results demonstrate differences between monolinguals and bilinguals in auditory brainstem responses elicited by speech in noise, even in the selected sample of normal-hearing young adults. This finding highlights the importance of considering an individual’s language background when assessing brain processing and listening skills. Following the current findings, we expect more pronounced differences in bilingual clinical populations with more deficient perceptual abilities (for example, hearing-impaired individuals or older adults). Future studies are needed to evaluate this issue. Further, by establishing relationships between brainstem processing and perception of speech in noise among bilinguals, the current study provides new insights into how bilingualism shapes the brain and the impact this has on everyday listening situations. Our results can help determine which individuals may have a biological signature for excessive difficulty in challenging listening conditions. These findings can aid in the development of programs to help individuals predicted to encounter more difficulties in challenging listening conditions. Perhaps bilinguals with a brainstem system that is more susceptible to noise effects could benefit from speech-in-noise training or from using assistive listening devices that enhance the signal-to-noise ratio in academic settings.

Future studies are needed to determine the generalizability of the findings while considering the current study’s limitations. In this study, perceptual and physiological stimuli were administered differently (binaurally versus monaurally and in the presence of white noise versus babble noise). In the physiological part, we followed previous protocols that presented the speech stimulus monaurally (e.g., [24, 25, 54, 58]). The monaural stimulation enables one ear to be unoccluded, maximizing participants’ alertness and promoting stillness. In addition, the use of a continuous, stable masker was preferred in the physiological part (similar to [20, 30, 159] using the 40 ms /da/] because we used a very short stimulus that made it difficult to combine with a constantly changing masker. Therefore, a future study that uses the same stimulation conditions in perceptual and neural measures is needed. Furthermore, as some differences between our findings and previous ones have been suggested to be attributed to using a shorter syllable, a follow-up study should examine the effects of stimulus duration and characteristics on physiological results. Such a study is critical to examine whether physiological differences observed depend on the characteristics of the speech being tested.

## Conclusions

The current study addresses the subcortical neural aspects underlying speech perception in noise among bilinguals. We provide evidence for differences between monolinguals and bilinguals in perceptual performance and auditory brainstem responses evoked by speech stimuli presented in challenging listening conditions. The current results show that the susceptibility of the auditory system at subcortical levels correlates with the ability of bilinguals to perceive speech presented in noise and can predict which individuals may be more prone to the detrimental effect of noise. Implications of the current results were discussed, and future studies were proposed.

## References

[1] Mattys SL, Davis MH, Bradlow AR, Scott SK. Speech recognition in adverse conditions: A review. Language and Cognitive Processes. 2012;27(7–8):953–978.

[2] Houtgast T. The effect of ambient noise on speech intelligibility in classrooms. Applied Acoustics.1981;14(1):15–25.

[3] Klatte M, Lachmann T, Meis M. Effects of noise and reverberation on speech perception and listening comprehension of children and adults in a classroom-like setting. Noise and Health. 2010;12(49):270. doi: 10.4103/1463-1741.70506 20871182

[4] Lecumberri MLG, Cooke M, Cutler A. Non-native speech perception in adverse conditions: A review. Speech communication. 2010;52(11–12):864–886.

[5] Borghini G, Hazan V. Listening effort during sentence processing is increased for non-native listeners: A pupillometry study. Frontiers in neuroscience. 2018;12:152. doi: 10.3389/fnins.2018.00152 29593489 PMC5859302

[6] Borghini G, Hazan V. Effects of acoustic and semantic cues on listening effort during native and non-native speech perception. The Journal of the Acoustical Society of America. 2020;147(6):3783–3794. doi: 10.1121/10.0001126 32611155

[7] Mayo LH, Florentine M, Buus S. Age of second-language acquisition and perception of speech in noise. Journal of speech, language, and hearing research. 1997;40(3):686–693. doi: 10.1044/jslhr.4003.686 9210123

[8] Morini G, Newman RS. Monolingual and Bilingual Word Recognition and Word Learning in Background Noise. Language and speech. 2020;63(2):381–403. doi: 10.1177/0023830919846158 31106697 PMC6861599

[9] Peng ZE, Wang LM. Listening effort by native and nonnative listeners due to noise, reverberation, and talker foreign accent during english speech perception. Journal of Speech, Language, and Hearing Research. 2019;62(4):1068–1081. doi: 10.1044/2018_JSLHR-H-17-0423 30986135

[10] Rogers CL, Lister JJ, Febo DM, Besing JM, Abrams HB. Effects of bilingualism, noise, and reverberation on speech perception by listeners with normal hearing. Applied Psycholinguistics. 2006;27(3):465.

[11] Scharenborg O, Coumans JM, van Hout R. The effect of background noise on the word activation process in nonnative spoken-word recognition. Journal of Experimental Psychology: Learning, Memory, and Cognition. 2018;44(2):233. doi: 10.1037/xlm0000441 28782967

[12] Tabri D, Chacra KMSA, Pring T. Speech perception in noise by monolingual, bilingual and trilingual listeners. International Journal of Language & Communication Disorders. 2015:1–12. 21771217 10.3109/13682822.2010.519372

[13] Scharenborg O, van Os M. Why listening in background noise is harder in a non-native language than in a native language: A review. Speech Communication. 2019;108:53–64.

[14] Rosenhouse J, Haik L, Kishon-Rabin L. Speech perception in adverse listening conditions in Arabic-Hebrew bilinguals. International Journal of Bilingualism. 2006;10(2):119–135.

[15] Florentine M, Buus S, Scharf B, Canevet G. Speech reception thresholds in noise for native and non-native listeners. The Journal of the Acoustical Society of America. 1984;75(S1):S84–S84.

[16] Bsharat-Maalouf D, Karawani H. Learning and bilingualism in challenging listening conditions: How challenging can it be? Cognition. 2022;222:105018. doi: 10.1016/j.cognition.2022.105018 35032867

[17] Skoe E, Karayanidi K. Bilingualism and speech understanding in noise: Auditory and linguistic factors. Journal of the American Academy of Audiology. 2019;30(02):115–130. doi: 10.3766/jaaa.17082 30461397

[18] Hayakawa S, Marian V. Consequences of multilingualism for neural architecture. Behavioral and Brain Functions. 2019;15(1):1–24. doi: 10.1186/s12993-019-0152-4 30909931 PMC6432751

[19] Bidelman GM. Communicating in challenging environments: noise and reverberation. The frequency-following response: Springer; 2017. p. 193–224.

[20] Koravand A, Thompson J, Chénier G, Kordjazi N. The effects of bilingualism on speech evoked brainstem responses recorded in quiet and in noise. Canadian Acoustics. 2019;47(2):23–30.

[21] Omote A, Jasmin K, Tierney A. Successful non-native speech perception is linked to frequency following response phase consistency. cortex. 2017;93:146–154. doi: 10.1016/j.cortex.2017.05.005 28654816 PMC5542039

[22] Giroud N, Baum SR, Gilbert AC, Phillips NA, Gracco V. Earlier age of second language learning induces more robust speech encoding in the auditory brainstem in adults, independent of amount of language exposure during early childhood. Brain and Language. 2020;207:104815. doi: 10.1016/j.bandl.2020.104815 32535187

[23] Maggu AR, Zong W, Law V, Wong PC, editors. Learning Two Tone Languages Enhances the Brainstem Encoding of Lexical Tones. INTERSPEECH; 2018. doi: 10.1016/j.mri.2017.09.011

[24] Krizman J, Marian V, Shook A, Skoe E, Kraus N. Subcortical encoding of sound is enhanced in bilinguals and relates to executive function advantages. Proceedings of the National Academy of Sciences. 2012;109(20):7877–7881. doi: 10.1073/pnas.1201575109 22547804 PMC3356657

[25] Krizman J, Skoe E, Marian V, Kraus N. Bilingualism increases neural response consistency and attentional control: Evidence for sensory and cognitive coupling. Brain and language. 2014;128(1):34–40. doi: 10.1016/j.bandl.2013.11.006 24413593 PMC3923605

[26] Skoe E, Burakiewicz E, Figueiredo M, Hardin M. Basic neural processing of sound in adults is influenced by bilingual experience. Neuroscience. 2017;349:278–290. doi: 10.1016/j.neuroscience.2017.02.049 28259798

[27] Krizman J, Slater J, Skoe E, Marian V, Kraus N. Neural processing of speech in children is influenced by extent of bilingual experience. Neuroscience letters. 2015;585:48–53. doi: 10.1016/j.neulet.2014.11.011 25445377 PMC4272867

[28] Krizman J, Skoe E, Kraus N. Bilingual enhancements have no socioeconomic boundaries. Developmental Science. 2016;19(6):881–891. doi: 10.1111/desc.12347 26573107

[29] Chandrasekaran B, Kraus N. The scalp-recorded brainstem response to speech: Neural origins and plasticity. Psychophysiology. 2010;47(2):236–246. doi: 10.1111/j.1469-8986.2009.00928.x 19824950 PMC3088516

[30] Russo N, Nicol T, Musacchia G, Kraus N. Brainstem responses to speech syllables. Clinical Neurophysiology. 2004;115(9):2021–2030. doi: 10.1016/j.clinph.2004.04.003 15294204 PMC2529166

[31] Skoe E, Kraus N. Auditory brainstem response to complex sounds: a tutorial. Ear and hearing. 2010;31(3):302. doi: 10.1097/AUD.0b013e3181cdb272 20084007 PMC2868335

[32] Krizman J, Kraus N. Analyzing the FFR: A tutorial for decoding the richness of auditory function. Hearing research. 2019;382:107779. doi: 10.1016/j.heares.2019.107779 31505395 PMC6778514

[33] Krizman J, Tierney A, Nicol T, Kraus N. Listening in the Moment: How Bilingualism Interacts With Task Demands to Shape Active Listening. Frontiers in neuroscience. 2021;15. doi: 10.3389/fnins.2021.717572 34955707 PMC8702653

[34] Anderson S, Skoe E, Chandrasekaran B, Zecker S, Kraus N. Brainstem correlates of speech-in-noise perception in children. Hearing research. 2010;270(1–2):151–157. doi: 10.1016/j.heares.2010.08.001 20708671 PMC2997182

[35] Song JH, Skoe E, Banai K, Kraus N. Perception of speech in noise: neural correlates. Journal of cognitive neuroscience. 2011;23(9):2268–2279. doi: 10.1162/jocn.2010.21556 20681749 PMC3253852

[36] Brokx JPL, Nooteboom SG. Intonation and the perceptual separation of simultaneous voices. Journal of Phonetics. 1982;10(1):23–36.

[37] Bird J, Darwin C. Effects of a difference in fundamental frequency in separating two sentences. Psychophysical and physiological advances in hearing. 1998:263–269.

[38] Darwin CJ, Brungart DS, Simpson BD. Effects of fundamental frequency and vocal-tract length changes on attention to one of two simultaneous talkers. The Journal of the Acoustical Society of America. 2003;114(5):2913–2922. doi: 10.1121/1.1616924 14650025

[39] Wechsler D. WAiS-iii: Psychological Corporation San Antonio, TX; 1997.

[40] ANSI. Specifications for Audiometers (ANSI S3.6–1989). New York: American National Standards Institute. 1989.

[41] Hall JW. Handbook of auditory evoked responses: Allyn & Bacon; 1992.

[42] Musacchia G, Strait D, Kraus N. Relationships between behavior, brainstem and cortical encoding of seen and heard speech in musicians and non-musicians. Hearing research. 2008;241(1–2):34–42. doi: 10.1016/j.heares.2008.04.013 18562137 PMC2701624

[43] Chandrasekaran B, Krishnan A, Gandour JT. Relative influence of musical and linguistic experience on early cortical processing of pitch contours. Brain and language. 2009;108(1):1–9. doi: 10.1016/j.bandl.2008.02.001 18343493 PMC2670545

[44] Musacchia G, Sams M, Skoe E, Kraus N. Musicians have enhanced subcortical auditory and audiovisual processing of speech and music. Proceedings of the National Academy of Sciences. 2007;104(40):15894–15898. 10.1073/pnas.0701498104 PMC2000431 17898180

[45] Parbery-Clark A, Skoe E, Kraus N. Musical experience limits the degradative effects of background noise on the neural processing of sound. Journal of Neuroscience. 2009;29(45):14100–14107. doi: 10.1523/JNEUROSCI.3256-09.2009 19906958 PMC6665054

[46] Wong PC, Skoe E, Russo NM, Dees T, Kraus N. Musical experience shapes human brainstem encoding of linguistic pitch patterns. Nature neuroscience. 2007;10(4):420–422. doi: 10.1038/nn1872 17351633 PMC4508274

[47] Bidelman GM, Krishnan A. Effects of reverberation on brainstem representation of speech in musicians and non-musicians. Brain research. 2010;1355:112–125. doi: 10.1016/j.brainres.2010.07.100 20691672 PMC2939203

[48] Strait DL, Parbery-Clark A, O’Connell S, Kraus N. Biological impact of preschool music classes on processing speech in noise. Developmental cognitive neuroscience. 2013;6:51–60. doi: 10.1016/j.dcn.2013.06.003 23872199 PMC3844086

[49] Weiss MW, Bidelman GM. Listening to the brainstem: musicianship enhances intelligibility of subcortical representations for speech. Journal of Neuroscience. 2015;35(4):1687–1691. doi: 10.1523/JNEUROSCI.3680-14.2015 25632143 PMC6795259

[50] White-Schwoch T, Carr KW, Anderson S, Strait DL, Kraus N. Older adults benefit from music training early in life: biological evidence for long-term training-driven plasticity. Journal of Neuroscience. 2013;33(45):17667–17674. doi: 10.1523/JNEUROSCI.2560-13.2013 24198359 PMC3818545

[51] Marian V, Blumenfeld HK, Kaushanskaya M. The Language Experience and Proficiency Questionnaire (LEAP-Q): Assessing language profiles in bilinguals and multilinguals. Journal of speech, language, and hearing research. 2007. doi: 10.1044/1092-4388(2007/067) 17675598

[52] Maddieson I, Disner SF. Patterns of sounds: Cambridge university press; 1984.

[53] Karawani H, Banai K. Speech-evoked brainstem responses in Arabic and Hebrew speakers. International journal of audiology. 2010;49(11):844–849. doi: 10.3109/14992027.2010.495083 20666694

[54] Krizman J, Skoe E, Kraus N. Stimulus rate and subcortical auditory processing of speech. Audiology and Neurotology. 2010;15(5):332–342. doi: 10.1159/000289572 20215743 PMC2919427

[55] Gorga M, Abbas P, Worthington D. Stimulus calibration in ABR measurements. The auditory brainstem response. 1985:49–62.

[56] Liu L-F, Palmer AR, Wallace MN. Phase-locked responses to pure tones in the inferior colliculus. Journal of neurophysiology. 2006;95(3):1926–1935. doi: 10.1152/jn.00497.2005 16339005

[57] Johnson KL, Nicol TG, Kraus N. Brain stem response to speech: a biological marker of auditory processing. Ear and hearing. 2005;26(5):424–434. doi: 10.1097/01.aud.0000179687.71662.6e 16230893

[58] Johnson KL, Nicol T, Zecker SG, Kraus N. Developmental plasticity in the human auditory brainstem. Journal of Neuroscience. 2008;28(15):4000–4007. doi: 10.1523/JNEUROSCI.0012-08.2008 18400899 PMC2806643

[59] Ratcliff ER. Psychometrically equivalent bisyllabic words for speech reception threshold testing in Arabic. 2006.

[60] Hagerman B. Sentences for testing speech intelligibility in noise. Scandinavian audiology. 1982;11(2):79–87. doi: 10.3109/01050398209076203 7178810

[61] Akker E, Cutler A. Prosodic cues to semantic structure in native and nonnative listening. Bilingualism: Language and Cognition. 2003;6(2):81–96.

[62] Kraus N, White-Schwoch T. The bilingualism paradox. The Hearing Journal. 2017;70(1):40–42.

[63] Lotfi Y, Chupani J, Javanbakht M, Bakhshi E. Evaluation of speech perception in noise in Kurd-Persian bilinguals. Auditory and Vestibular Research. 2019;28(1):36–41.

[64] Krizman J, Bradlow AR, Lam SS-Y, Kraus N. How bilinguals listen in noise: Linguistic and non-linguistic factors. Bilingualism: Language and Cognition. 2017;20(4):834–843.

[65] Kaan E. Predictive sentence processing in L2 and L1: What is different? Linguistic Approaches to Bilingualism. 2014;4(2):257–282.

[66] Martin CD, Thierry G, Kuipers J-R, Boutonnet B, Foucart A, Costa A. Bilinguals reading in their second language do not predict upcoming words as native readers do. Journal of Memory and Language. 2013;69(4):574–588.

[67] Lucks Mendel L, Widner H. Speech perception in noise for bilingual listeners with normal hearing. International Journal of Audiology. 2016;55(2):126–134. doi: 10.3109/14992027.2015.1061710 26189557

[68] Bradlow AR, Blasingame M, Lee K. Language-independent talker-specificity in bilingual speech intelligibility: Individual traits persist across first-language and second-language speech. Laboratory Phonology: Journal of the Association for Laboratory Phonology. 2018;9(1).

[69] Karawani H, Bitan T, Attias J, Banai K. Auditory perceptual learning in adults with and without age-related hearing loss. Frontiers in psychology. 2016;6:2066. doi: 10.3389/fpsyg.2015.02066 26869944 PMC4737899

[70] Abdi H. Bonferroni and Šidák corrections for multiple comparisons. Encyclopedia of measurement and statistics. 2007;3:103–107.

[71] Anderson S, Parbery-Clark A, White-Schwoch T, Kraus N. Aging affects neural precision of speech encoding. Journal of Neuroscience. 2012;32(41):14156–14164. doi: 10.1523/JNEUROSCI.2176-12.2012 23055485 PMC3488287

[72] Coffey EB, Chepesiuk AM, Herholz SC, Baillet S, Zatorre RJ. Neural correlates of early sound encoding and their relationship to speech-in-noise perception. Frontiers in neuroscience. 2017;11:479. doi: 10.3389/fnins.2017.00479 28890684 PMC5575455

[73] Du Y, Kong L, Wang Q, Wu X, Li L. Auditory frequency-following response: a neurophysiological measure for studying the “cocktail-party problem”. Neuroscience & Biobehavioral Reviews. 2011;35(10):2046–2057. doi: 10.1016/j.neubiorev.2011.05.008 21645541

[74] Anderson S, Skoe E, Chandrasekaran B, Kraus N. Neural timing is linked to speech perception in noise. Journal of Neuroscience. 2010;30(14):4922–4926. doi: 10.1523/JNEUROSCI.0107-10.2010 20371812 PMC2862599

[75] Anderson S, Kraus N. Sensory-cognitive interaction in the neural encoding of speech in noise: a review. Journal of the American Academy of Audiology. 2010;21(09):575–585. doi: 10.3766/jaaa.21.9.3 21241645 PMC3075209

[76] Parbery-Clark A, Marmel F, Bair J, Kraus N. What subcortical–cortical relationships tell us about processing speech in noise. European Journal of Neuroscience. 2011;33(3):549–557. doi: 10.1111/j.1460-9568.2010.07546.x 21255123

[77] Song JH, Nicol T, Kraus N. Test–retest reliability of the speech-evoked auditory brainstem response. Clinical Neurophysiology. 2011;122(2):346–355. doi: 10.1016/j.clinph.2010.07.009 20719558 PMC2990784

[78] Desjardins JL, Barraza EG, Orozco JA. Age-related changes in speech recognition performance in Spanish–English bilinguals’ first and second languages. Journal of Speech, Language, and Hearing Research. 2019;62(7):2553–2563. doi: 10.1044/2019_JSLHR-H-18-0435 31251686 PMC6808351

[79] Garcia Lecumberri MLG, Cooke M, Cutler A. Non-native speech perception in adverse conditions: A review. Speech communication. 2010;52(11–12):864–886.

[80] Bidelman GM, Dexter L. Bilinguals at the “cocktail party”: Dissociable neural activity in auditory–linguistic brain regions reveals neurobiological basis for nonnative listeners’ speech-in-noise recognition deficits. Brain and language. 2015;143:32–41. doi: 10.1016/j.bandl.2015.02.002 25747886

[81] Bradlow AR, Alexander JA. Semantic and phonetic enhancements for speech-in-noise recognition by native and non-native listeners. The Journal of the Acoustical Society of America. 2007;121(4):2339–2349. doi: 10.1121/1.2642103 17471746

[82] Meador D, Flege JE, MacKay IR. Factors affecting the recognition of words in a second language. Bilingualism: Language and Cognition. 2000;3(1):55–67.

[83] Hurtig A, Keus van de Poll M, Pekkola EP, Hygge S, Ljung R, Sörqvist P. Children’s recall of words spoken in their first and second language: effects of signal-to-noise ratio and reverberation time. Frontiers in psychology. 2016;6:2029. doi: 10.3389/fpsyg.2015.02029 26834665 PMC4712295

[84] Hygge S, Kjellberg A, Nöstl A. Speech intelligibility and recall of first and second language words heard at different signal-to-noise ratios. Frontiers in Psychology. 2015;6:1390. doi: 10.3389/fpsyg.2015.01390 26441765 PMC4568391

[85] Weiss D, Dempsey JJ. Performance of bilingual speakers on the English and Spanish versions of the Hearing in Noise Test (HINT). Journal of the American Academy of Audiology. 2008;19(1):5–17. doi: 10.3766/jaaa.19.1.2 18637406

[86] Ezzatian P, Avivi M, Schneider BA. Do nonnative listeners benefit as much as native listeners from spatial cues that release speech from masking? Speech Communication. 2010;52(11–12):919–929.

[87] Sorace A. Incomplete vs. divergent representations of unaccusativity in non native grammars of Italian. Second Language Research. 1993;9(1):22–47.

[88] Kilman L, Zekveld A, Hällgren M, Rönnberg J. The influence of non-native language proficiency on speech perception performance. Frontiers in Psychology. 2014;5:651. doi: 10.3389/fpsyg.2014.00651 25071630 PMC4078910

[89] Gollan TH, Slattery TJ, Goldenberg D, Van Assche E, Duyck W, Rayner K. Frequency drives lexical access in reading but not in speaking: The frequency-lag hypothesis. Journal of Experimental Psychology: General. 2011;140(2):186. 21219080 10.1037/a0022256 PMC3086969

[90] Gollan TH, Montoya RI, Cera C, Sandoval TC. More use almost always means a smaller frequency effect: Aging, bilingualism, and the weaker links hypothesis. Journal of memory and language. 2008;58(3):787–814. doi: 10.1016/j.jml.2007.07.001 19343088 PMC2409197

[91] Schmidtke J. The bilingual disadvantage in speech understanding in noise is likely a frequency effect related to reduced language exposure. Frontiers in Psychology. 2016;7:678. doi: 10.3389/fpsyg.2016.00678 27242592 PMC4865492

[92] Degani T, Prior A, Tokowicz N. Bidirectional transfer: The effect of sharing a translation. Journal of Cognitive Psychology. 2011;23(1):18–28.

[93] Weber A, Cutler A. Lexical competition in non-native spoken-word recognition. Journal of Memory and Language. 2004;50(1):1–25.

[94] Hermans D, Bongaerts T, De Bot K, Schreuder R. Producing words in a foreign language: Can speakers prevent interference from their first language? Bilingualism: language and cognition. 1998;1(3):213–229.

[95] Shook A, Marian V. The bilingual language interaction network for comprehension of speech. Bilingualism: Language and Cognition. 2013;16(2):304–324. 10.1017/S1366728912000466 PMC3866103 24363602

[96] Marian V, Spivey M. Competing activation in bilingual language processing: Within-and between-language competition. Bilingualism: Language and cognition. 2003;6(2):97–115.

[97] BinKhamis G, Léger A, Bell SL, Prendergast G, O’Driscoll M, Kluk K. Speech auditory brainstem responses: Effects of background, stimulus duration, consonant–vowel, and number of epochs. Ear and hearing. 2019;40(3):659. doi: 10.1097/AUD.0000000000000648 30124503 PMC6493675

[98] Yellamsetty A, Bidelman GM. Brainstem correlates of concurrent speech identification in adverse listening conditions. Brain research. 2019;1714:182–192. doi: 10.1016/j.brainres.2019.02.025 30796895 PMC6727209

[99] Tierney A, Parbery-Clark A, Skoe E, Kraus N. Frequency-dependent effects of background noise on subcortical response timing. Hearing research. 2011;282(1–2):145–150. doi: 10.1016/j.heares.2011.08.014 21907782 PMC3230695

[100] Burkard RF, Sims D. A comparison of the effects of broadband masking noise on the auditory brainstem response in young and older adults. Young. 2002;40(50):60. doi: 10.1044/1059-0889(2002/004) 12227352

[101] Carcagno S, Plack CJ. Subcortical plasticity following perceptual learning in a pitch discrimination task. Journal of the Association for Research in Otolaryngology. 2011;12(1):89–100. doi: 10.1007/s10162-010-0236-1 20878201 PMC3015031

[102] Sanju HK, Kumar P. Enhanced auditory evoked potentials in musicians: A review of recent findings. Journal of Otology. 2016;11(2):63–72. doi: 10.1016/j.joto.2016.04.002 29937812 PMC6002589

[103] Krishnan A, Xu Y, Gandour J, Cariani P. Encoding of pitch in the human brainstem is sensitive to language experience. Cognitive Brain Research. 2005;25(1):161–168. doi: 10.1016/j.cogbrainres.2005.05.004 15935624

[104] Krishnan A, Xu Y, Gandour JT, Cariani PA. Human frequency-following response: representation of pitch contours in Chinese tones. Hearing research. 2004;189(1–2):1–12. doi: 10.1016/S0378-5955(03)00402-7 14987747

[105] Russo NM, Nicol TG, Zecker SG, Hayes EA, Kraus N. Auditory training improves neural timing in the human brainstem. Behavioural brain research. 2005;156(1):95–103. doi: 10.1016/j.bbr.2004.05.012 15474654

[106] Krishnamurti S, Forrester J, Rutledge C, Holmes GW. A case study of the changes in the speech-evoked auditory brainstem response associated with auditory training in children with auditory processing disorders. International journal of pediatric otorhinolaryngology. 2013;77(4):594–604. doi: 10.1016/j.ijporl.2012.12.032 23357780

[107] Filippini R, Befi-Lopes D, Schochat E. Efficacy of auditory training using the auditory brainstem response to complex sounds: auditory processing disorder and specific language impairment. Folia Phoniatrica et Logopaedica. 2012;64(5):217–226. doi: 10.1159/000342139 23006808

[108] Song JH, Skoe E, Wong PC, Kraus N. Plasticity in the adult human auditory brainstem following short-term linguistic training. Journal of cognitive neuroscience. 2008;20(10):1892–1902. doi: 10.1162/jocn.2008.20131 18370594 PMC2829864

[109] Adesope OO, Lavin T, Thompson T, Ungerleider C. A systematic review and meta-analysis of the cognitive correlates of bilingualism. Review of Educational Research. 2010;80(2):207–245.

[110] Bialystok E, Martin MM. Attention and inhibition in bilingual children: Evidence from the dimensional change card sort task. Developmental science. 2004;7(3):325–339. doi: 10.1111/j.1467-7687.2004.00351.x 15595373

[111] Bialystok E. Reshaping the mind: the benefits of bilingualism. Canadian Journal of Experimental Psychology/Revue canadienne de psychologie expérimentale. 2011;65(4):229. doi: 10.1037/a0025406 21910523 PMC4341987

[112] Blumenfeld HK, Marian V. Bilingualism influences inhibitory control in auditory comprehension. Cognition. 2011;118(2):245–257. doi: 10.1016/j.cognition.2010.10.012 21159332 PMC3582323

[113] Carlson SM, Meltzoff AN. Bilingual experience and executive functioning in young children. Developmental science. 2008;11(2):282–298. doi: 10.1111/j.1467-7687.2008.00675.x 18333982 PMC3647884

[114] Grote KS, Scott RM, Gilger J. Bilingual advantages in executive functioning: Evidence from a low-income sample. First Language. 2021:01427237211024220.

[115] Soveri A, Laine M, Hämäläinen H, Hugdahl K. Bilingual advantage in attentional control: Evidence from the forced-attention dichotic listening paradigm. Bilingualism: Language and Cognition. 2011;14(3):371–378.

[116] Ruggles D, Bharadwaj H, Shinn-Cunningham BG. Normal hearing is not enough to guarantee robust encoding of suprathreshold features important in everyday communication. Proceedings of the National Academy of Sciences. 2011;108(37):15516–15521. doi: 10.1007/s10162-010-0254-z 21844339 PMC3174666

[117] Hairston WD, Letowski TR, McDowell K. Task-related suppression of the brainstem frequency following response. PLoS One. 2013;8(2):e55215. doi: 10.1371/journal.pone.0055215 23441150 PMC3575437

[118] Raizada RD, Poldrack RA. Challenge-driven attention: Interacting frontal and brainstem systems. Frontiers in human neuroscience. 2008;2:3. doi: 10.3389/neuro.09.003.2008 18958217 PMC2525983

[119] Rinne T, Balk MH, Koistinen S, Autti T, Alho K, Sams M. Auditory selective attention modulates activation of human inferior colliculus. Journal of Neurophysiology. 2008;100(6):3323–3327. doi: 10.1152/jn.90607.2008 18922948

[120] Bidelman GM, Pousson M, Dugas C, Fehrenbach A. Test–retest reliability of dual-recorded brainstem versus cortical auditory-evoked potentials to speech. Journal of the American Academy of Audiology. 2018;29(02):164–174. 29401063 10.3766/jaaa.16167

[121] Ahissar M, Hochstein S. The reverse hierarchy theory of visual perceptual learning. Trends in cognitive sciences. 2004;8(10):457–464. doi: 10.1016/j.tics.2004.08.011 15450510

[122] Grundy JG, Anderson JA, Bialystok E. Neural correlates of cognitive processing in monolinguals and bilinguals. Annals of the New York Academy of Sciences. 2017;1396(1):183. doi: 10.1111/nyas.13333 28415142 PMC5446278

[123] Kraus N. Memory for sound: the BEAMS hypothesis [Perspective]. Hearing research. 2021;407(10829):1. 10.1016/j.heares.2021.108291 34146833

[124] Bauer LO, Bayles RL. Precortical filtering and selective attention: an evoked potential analysis. Biological Psychology. 1990;30(1):21–33. doi: 10.1016/0301-0511(90)90088-e 2223933

[125] Galbraith GC, Bhuta SM, Choate AK, Kitahara JM, Mullen TA Jr. Brain stem frequency-following response to dichotic vowels during attention. Neuroreport. 1998;9(8):1889–1893. doi: 10.1097/00001756-199806010-00041 9665621

[126] Galbraith GC, Jhaveri SP, Kuo J. Speech-evoked brainstem frequency-following responses during verbal transformations due to word repetition. Electroencephalography and clinical neurophysiology. 1997;102(1):46–53. doi: 10.1016/s0013-4694(96)96006-x 9060854

[127] Lukas JH. The role of efferent inhibition in human auditory attention: an examination of the auditory brainstem potentials. International Journal of Neuroscience. 1981;12(2):137–145. doi: 10.3109/00207458108985796 7203823

[128] Song JH, Banai K, Kraus N. Brainstem timing deficits in children with learning impairment may result from corticofugal origins. Audiology and Neurotology. 2008;13(5):335–344. doi: 10.1159/000132689 18493120

[129] Bidelman GM, Davis MK, Pridgen MH. Brainstem-cortical functional connectivity for speech is differentially challenged by noise and reverberation. Hearing research. 2018;367:149–160. doi: 10.1016/j.heares.2018.05.018 29871826 PMC6105463

[130] Bidelman GM, Alain C. Hierarchical neurocomputations underlying concurrent sound segregation: connecting periphery to percept. Neuropsychologia. 2015;68:38–50. doi: 10.1016/j.neuropsychologia.2014.12.020 25542675

[131] Malmierca MS, Ryugo DK. Descending connections of auditory cortex to the midbrain and brain stem. The auditory cortex: Springer; 2011. p. 189–208.

[132] de Boer J, Thornton ARD. Neural correlates of perceptual learning in the auditory brainstem: efferent activity predicts and reflects improvement at a speech-in-noise discrimination task. Journal of Neuroscience. 2008;28(19):4929–4937. doi: 10.1523/JNEUROSCI.0902-08.2008 18463246 PMC6670751

[133] Gao E, Suga N. Experience-dependent plasticity in the auditory cortex and the inferior colliculus of bats: role of the corticofugal system. Proceedings of the National Academy of Sciences. 2000;97(14):8081–8086. doi: 10.1073/pnas.97.14.8081 10884432 PMC16673

[134] Lotfi Y, Moossavi A, Javanbakht M, Zadeh SF. Speech-ABR in contralateral noise: A potential tool to evaluate rostral part of the auditory efferent system. Medical hypotheses. 2019;132:109355. doi: 10.1016/j.mehy.2019.109355 31604162

[135] Nahum M, Nelken I, Ahissar M. Low-level information and high-level perception: the case of speech in noise. PLoS biology. 2008;6(5):e126. doi: 10.1371/journal.pbio.0060126 18494561 PMC2386842

[136] Malmierca MS, Anderson LA, Antunes FM. The cortical modulation of stimulus-specific adaptation in the auditory midbrain and thalamus: a potential neuronal correlate for predictive coding. Frontiers in systems neuroscience. 2015;9:19. doi: 10.3389/fnsys.2015.00019 25805974 PMC4353371

[137] Bishop CW, Miller LM. A multisensory cortical network for understanding speech in noise. Journal of cognitive neuroscience. 2009;21(9):1790–1804. doi: 10.1162/jocn.2009.21118 18823249 PMC2833290

[138] Gutschalk A, Micheyl C, Oxenham AJ. Neural correlates of auditory perceptual awareness under informational masking. PLoS biology. 2008;6(6):e138. doi: 10.1371/journal.pbio.0060138 18547141 PMC2422852

[139] Obleser J, Wise RJ, Dresner MA, Scott SK. Functional integration across brain regions improves speech perception under adverse listening conditions. Journal of Neuroscience. 2007;27(9):2283–2289. doi: 10.1523/JNEUROSCI.4663-06.2007 17329425 PMC6673469

[140] Scott SK, Rosen S, Beaman CP, Davis JP, Wise RJ. The neural processing of masked speech: Evidence for different mechanisms in the left and right temporal lobes. The Journal of the Acoustical Society of America. 2009;125(3):1737–1743. doi: 10.1121/1.3050255 19275330

[141] Scott SK, Rosen S, Wickham L, Wise RJ. A positron emission tomography study of the neural basis of informational and energetic masking effects in speech perception. The Journal of the Acoustical Society of America. 2004;115(2):813–821. doi: 10.1121/1.1639336 15000192

[142] Wong PC, Uppunda AK, Parrish TB, Dhar S. Cortical mechanisms of speech perception in noise. 2008. 10.1044/1092-4388(2008/075) 18658069

[143] Zekveld AA, Heslenfeld DJ, Festen JM, Schoonhoven R. Top–down and bottom–up processes in speech comprehension. Neuroimage. 2006;32(4):1826–1836. doi: 10.1016/j.neuroimage.2006.04.199 16781167

[144] Suga N, Gao E, Zhang Y, Ma X, Olsen JF. The corticofugal system for hearing: recent progress. Proceedings of the National Academy of Sciences. 2000;97(22):11807–11814. doi: 10.1073/pnas.97.22.11807 11050213 PMC34353

[145] Zhang Y, Suga N. Corticofugal feedback for collicular plasticity evoked by electric stimulation of the inferior colliculus. Journal of Neurophysiology. 2005;94(4):2676–2682. doi: 10.1152/jn.00549.2005 16000518

[146] Hervais-Adelman A, Egorova N, Golestani N. Beyond bilingualism: multilingual experience correlates with caudate volume. Brain Structure and Function. 2018;223(7):3495–3502. doi: 10.1007/s00429-018-1695-0 29948191

[147] Zou L, Ding G, Abutalebi J, Shu H, Peng D. Structural plasticity of the left caudate in bimodal bilinguals. Cortex. 2012;48(9):1197–1206. doi: 10.1016/j.cortex.2011.05.022 21741636

[148] Olulade O, Jamal N, Koo D, Perfetti C, LaSasso C, Eden G. Neuroanatomical evidence in support of the bilingual advantage theory. Cerebral Cortex. 2015;26(7):3196–3204. doi: 10.1093/cercor/bhv152 26184647 PMC4898671

[149] Abutalebi J, Guidi L, Borsa V, Canini M, Della Rosa PA, Parris BA, et al. Bilingualism provides a neural reserve for aging populations. Neuropsychologia. 2015;69:201–210. doi: 10.1016/j.neuropsychologia.2015.01.040 25637228

[150] Perrone-Bertolotti M, Tassin M, Meunier F. Speech-in-speech perception and executive function involvement. PLoS One. 2017;12(7):e0180084. doi: 10.1371/journal.pone.0180084 28708830 PMC5510830

[151] Rudner M, Signoret C. The role of working memory and executive function in communication under adverse conditions. Frontiers in psychology. 2016;7:148. doi: 10.3389/fpsyg.2016.00148 26903938 PMC4749708

[152] Tremblay A, Namjoshi J, Spinelli E, Broersma M, Cho T, Kim S, et al. Experience with a second language affects the use of fundamental frequency in speech segmentation. PloS one. 2017;12(7):e0181709. doi: 10.1371/journal.pone.0181709 28738093 PMC5524284

[153] Assmann P, Summerfield Q. The perception of speech under adverse conditions. Speech processing in the auditory system: Springer; 2004. p. 231–308.

[154] Grüter T, Lew-Williams C, Fernald A. Grammatical gender in L2: A production or a real-time processing problem? Second Language Research. 2012;28(2):191–215. doi: 10.1177/0267658312437990 30319164 PMC6181447

[155] Warzybok A, Brand T, Wagener KC, Kollmeier B. How much does language proficiency by non-native listeners influence speech audiometric tests in noise? International journal of audiology. 2015;54(sup2):88–99. doi: 10.3109/14992027.2015.1063715 26344170

[156] Hopp H. Grammatical gender in adult L2 acquisition: Relations between lexical and syntactic variability. Second Language Research. 2013;29(1):33–56.

[157] Rönnberg J, Holmer E, Rudner M. Cognitive hearing science and ease of language understanding. International Journal of Audiology. 2019;58(5):247–261. doi: 10.1080/14992027.2018.1551631 30714435

[158] Rönnberg J, Lunner T, Zekveld A, Sörqvist P, Danielsson H, Lyxell B, et al. The Ease of Language Understanding (ELU) model: theoretical, empirical, and clinical advances. Frontiers in systems neuroscience. 2013;7:31. doi: 10.3389/fnsys.2013.00031 23874273 PMC3710434

[159] Anderson S, Parbery-Clark A, White-Schwoch T, Kraus N. Auditory brainstem response to complex sounds predicts self-reported speech-in-noise performance. 2013. 10.1044/1092-4388(2012/12-0043) PMC3648418 22761320

